# Diffuse planar xanthomatosis in the setting of monoclonal gammopathy of undetermined significance

**DOI:** 10.1111/1346-8138.17432

**Published:** 2024-08-21

**Authors:** Marisa Lenga, Jennifer Nam Choi

**Affiliations:** ^1^ Department of Dermatology Northwestern University, Feinberg School of Medicine Chicago Illinois USA; ^2^ Loyola University Chicago Stritch School of Medicine Maywood Illinois USA

**Keywords:** monoclonal gammomathy of underdermined significance, xanthomatosis

A woman in her 60s presented to the clinic for progressively accumulating, asymptomatic, yellow plaques for 6 years. Her medical history included monoclonal gammopathy of undetermined significance (MGUS) of IgG lambda light chain subtype for 6 years and hypercholesteremia. On physical examination, well‐defined, thin, yellow plaques were located on the eyelids, upper chest, anterior upper arms, axillae, and antecubital fossae without mucosal lesions (Figure 1). Histopathological examination revealed scattered collections of foamy histiocytes in the superficial and midreticular dermis with mild perivascular lymphohistiocytic infiltrate. Lipid panel findings demonstrated a mildly elevated low‐density lipoprotein cholesterol at 122 mg/dL (reference < 100 mg/dL). The patient was diagnosed with diffuse planar xanthomatosis in the setting of MGUS. Because the patient did not require treatment for MGUS at the time, skin‐directed treatment options were reviewed with the patient, and she elected to treat the left anterior arm with cryotherapy. She was lost to follow‐up.

**FIGURE 1:**
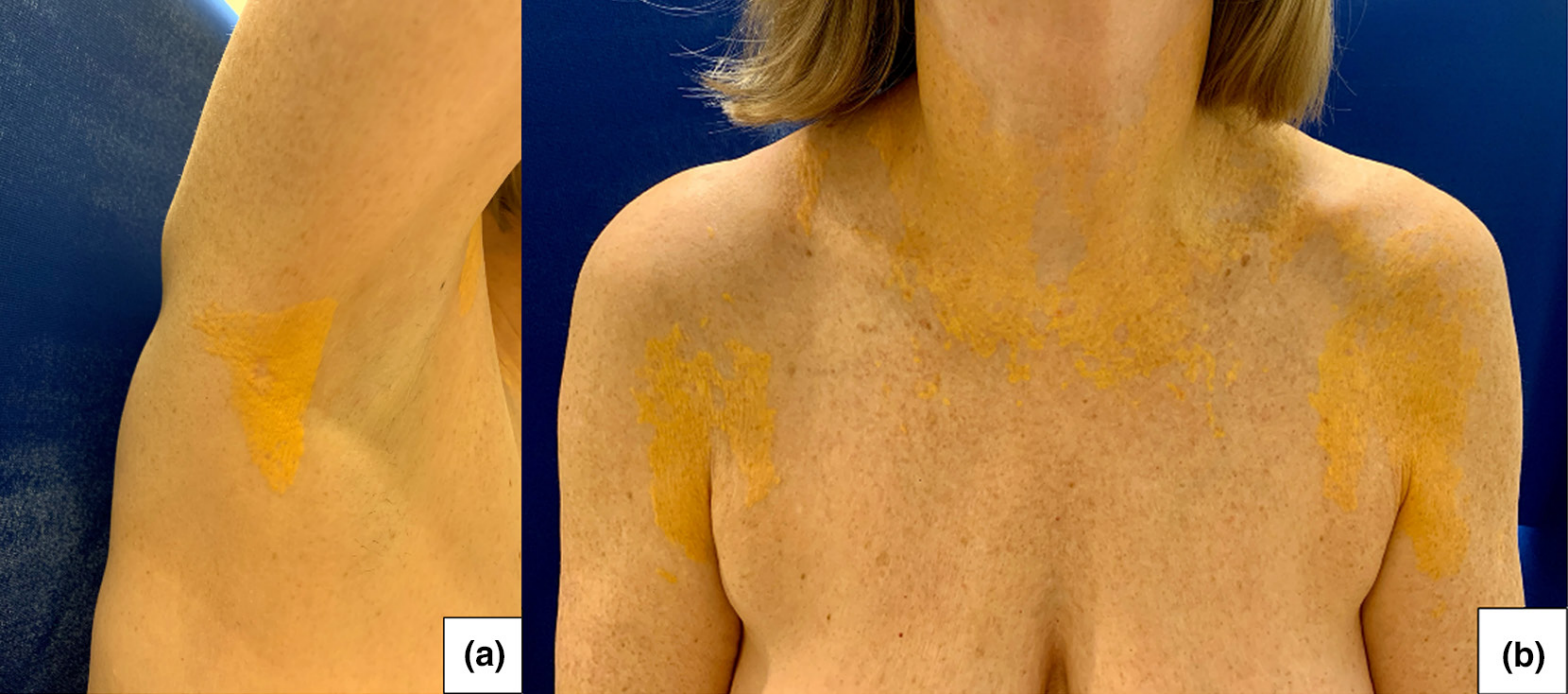
(a) Well‐defined triangular yellow plaques under right axilla. (b) Thin, yellow plaques across the neck and upper chest.

Diffuse planar xanthomatosis is characterized by the dispersed accumulation of cholesterol‐rich material in the skin and can develop in patients either with familial hyperlipidemia or with normal and slightly elevated lipids without family history.^1^, ^2^ In normolipidemic patients, there is a described phenomenon of diffuse xanthomas forming in the setting of hematological or lymphoproliferative disorders.^1^ Xanthomas form in the setting of MGUS (most commonly the IgG kappa subtype) through the creation of monoclonal IgG and low‐density lipoprotein complexes and the subsequent phagocytosis of these complexes by macrophages.^3^ This disease presents as asymptomatic, symmetric, yellow to orange plaques most commonly on the eyelids, neck, upper trunk, and flexural folds.^4^ Histopathologic examination demonstrates diffuse foamy cells in the dermis with variable numbers of giant cells, lymphocytes, and foamy histiocytes.^4^ Xanthomatosis may resolve spontaneously, with the treatment of MGUS, or with intervention like excision, chemabrasion, ablative laser, and cryotherapy.^3^, ^4^

This case serves as representative examination findings to improve clinical recognition of diffuse xanthomatosis in the setting of MGUS.

## CONFLICT OF INTEREST STATEMENT

The authors declare no conflicts of interest for this article.

## Data Availability

The principal investigator had full access to all of the data in the study and takes responsibility for the integrity of the data and the accuracy of the data analysis.
